# Phase Transformation Processes in Coprecipitated Cu/Zn/Zr Methanol Catalyst Precursors—Insights into Suspension Aging Form Ultrafast Nucleation

**DOI:** 10.1002/cplu.202500284

**Published:** 2025-07-09

**Authors:** Lucas Warmuth, Thomas A. Zevaco, David Guse, Michael Zimmermann, Matthias Kind, Stephan Pitter

**Affiliations:** ^1^ Institute of Catalysis Research and Technology (IKFT) Karlsruhe Institute of Technology (KIT) Hermann‐von‐Helmholtz‐Platz 1 76344 Eggenstein‐Leopoldshafen Germany; ^2^ Institute of Thermal Process Engineering (TVT) Karlsruhe Institute of Technology (KIT) Kaiserstraße 12 76131 Karlsruhe Germany

**Keywords:** coprecipitation, methanol catalysts, recrystallizations, suspension aging, zincian malachites

## Abstract

Catalyst precursors for methanol synthesis prepared by coprecipitation, such as Cu/Zn‐based hydroxycarbonates, are generally formed within two steps. The initial precipitation phase (nucleation) leads to a suspension from which, in a subsequent aging phase, the final solid precursor is formed, thus providing the structural and morphological features required for later use in catalysis. Combing ultrafast continuous nucleation with sampling from batch‐wise aging opens up the possibility to follow the evolution and transitions of solid phases during suspension aging. The temporal progression of the existence of the different phases in a Cu/ZnO/ZrO_2_‐based system is investigated by scanning electron microscopy, X‐ray diffraction, and inductive coupled plasma optical emission spectroscopy . According to the findings of this study, the intermediate recrystallization reveals to be a yet unknown two‐step process. The presence of an amorphous transient zinc depot of Na_2_Zn_3_(CO_3_)_4_ × 3 H_2_O greatly influences the formation of the relevant zincian malachite catalyst precursor [(Cu,Zn)_2_(OH)_2_CO_3_], which is partly in contrast to reports on Cu/ZnO/Al_2_O_3_ systems. Finally, a general mechanism including the relevant transformations during suspension aging in Cu/ZnO/ZrO_2_ systems is proposed, relying on a general thermodynamic approach, explaining the transient and final species.

## Graphical abstract

Catalyst production for methanol synthesis includes an aging step. By ultrafast nucleation, occurring transient changes in the suspension important for later performance can be assessed. Analytical techniques deliver insights which lead to a mechanism in which zincian malachite is the main product, whereas a sodium species play an important role as transient zinc depot.

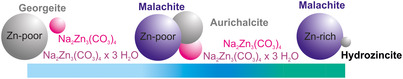

## 1 Introduction

Methanol is one of the key platform chemicals in chemical industry, also offering potential as chemical energy carrier or as vector for hydrogen storage. The potential of methanol is also reflected by its increasing global demand (2015: ≈75 Mt; 2020: ≈110 Mt; 2025 estimate: ≈145 Mt).^[^ ^1^ ^]^ For industrial methanol synthesis, performed using synthesis gas with a low CO_2_ content (<10%), Cu/ZnO/Al_2_O_3_ (CZA) catalysts are most commonly used. Fundamental characteristics of CZA catalysts have been widely studied and particular structure‐activity relationships have been gathered.^[^ ^2^ ^]^ In recent years, several investigations on the pivotal aging step have been published for Cu/ZnO (CZ‐) and CZA‐based systems.[^2^, ^3^] For example, the studies of Güldenpfennig et al. and Behrendt et al. provide detailed insights into aging mechanisms for CZ(Mg) systems.[^3^, ^4^] In a detailed study, Behrens et al.^[2b]^ show that several phases may emerge during aging of Cu/Zn‐based hydroxycarbonates (**Table** **1**).

**Table 1:** Cu/Zn hydroxycarbonate phases related to precipitation and subsequent aging. (Cu/Al–hydrotalcite formation is reported to be important for Cu/ZnO/Al_2_O_3_ (CZA) systems,^[3i]^ but no relevance has been shown for CZZ. Additionally, binary copper or zinc zirconates are unlikely to form in aqueous systems at typical conditions.[^2^, ^14^] the overall formula of GE is the topic of ongoing scientific discussion.^[^ ^23^ ^]^)

Formula	Geological name	Zinc content [mol% of Cu+Zn]	Abbreviation [this work]
(Cu, Zn)_2_(OH)_2_CO_3_	(Zincian) Malachite	0–31^[^ ^13^, ^14^ ^]^	MA
(Cu, Zn)_2_(OH)_2_CO_3_	Rosasite	30–50^[^ ^13^, ^14^ ^]^	RO
(Cu, Zn)_5_(OH)_4_(CO_3_)_3_ × 6 H_2_O	(Zincian) Georgeite	n. A. (0–36%?)^[^ ^23^ ^]^	GE
(Cu, Zn)_5_(OH)_6_(CO_3_)_2_	Aurichalcite	50–90^[^ ^24^ ^]^	AU
Zn_5_(OH)_6_(CO_3_)_2_	Hydrozincite	100	HZ
Na_2_Zn_3_(CO_3_)_4_	n. A.	100^[^ ^16^ ^]^	NaZC
Na_2_Zn_3_(CO_3_)_4_ × 3 H_2_O	n. A.	100^[^ ^16^ ^]^	NaZCH

According to the current state of research, the optimal target phase before calcination is the malachite (MA) type with maximum zinc enrichment [(Cu,Zn)_2_(OH)_2_CO_3_, Zn‐MA].[^3^, ^5^] Presence of Zn–MA ensures that, after the subsequent calcination and activation steps, generation of constructive Cu–Zn interactions is preferred. These interactions are expressed by the relative particle sizes of Cu and ZnO in the active (e.g., calcined and reduced) catalyst, leading to highly accessible active sites^[3b]^ indicated by high specific surface areas and active Cu surface areas.^[^ ^6^ ^]^

Cu/ZnO/ZrO_2_ (CZZ) catalysts are promising candidates to overcome these challenges, offering good productivity as well as acceptable stability with CO_2_‐rich feeds. They combine sufficiently high methanol productivity and long‐time stability at the thereby induced water‐rich conditions.^[^ ^7^ ^]^ Employing CO_2_ as sustainable chemical feedstock in methanol production, for example using industrial CO_2_ point sources, could pave the way to an improved sustainability. Although CZZ catalysts have been the subject of numerous studies dealing with catalytic applications and various approaches to catalyst synthesis, comparatively little is known about the aging processes in freshly precipitated CZZ suspensions. From our point of view, the identification of the influences of manufacturing parameters on the subsequent material properties is of primary importance, as well as their impact on a quality‐controlled and scalable catalyst production.

Recently, we reported on a modified coprecipitation approach to produce larger quantities of CZZ.^[^ ^8^ ^]^ Key step is the ultrafast (kinetically controlled) nucleation under highly turbulent conditions in a micromixer, continuously yielding amorphous precipitate at highly identical conditions ready for the subsequent aging.^[8b]^ As aging is reflecting the period within which thermodynamically controlled phase transformations occur, a detailed knowledge of the evolution of amorphous and crystalline phases is crucial for systematic optimization of aging parameters. This approach largely minimizes the temporal overlap of both processes as continuous nucleation is completed within less than 5 min and subsequent aging takes about 1 to 2 h.^[8a]^ This enables sampling of representative, homogeneous samples with a defined process time during aging not interfered from simultaneously occurring precipitation phenomena, as often encountered in the typically used, comparatively slow precipitation by dropwise addition of two stock solutions (e.g., involving metal nitrate and carbonate/bicarbonate salts).

Recently, we reported on indicators related to CZZ aging and related transient species, which are observed by in situ and ex situ Fourier transform infrared spectroscopy (FTIR).^[^ ^9^ ^]^ Incidentally, the presence of temporarily emerging crystal phases such as small amounts of aurichalcite (AU) in the early stages of the suspension aging are correlated to the so‐called pH‐tipping point. This short drop in pH accompanied by phase transformations is as also documented for CZA in the studies from Güldenpfennig and Behrendt et al.[^3^, ^4^, ^9^] Frei et al. reported how precipitation and aging temperatures are influencing the properties of final, dry CZZ catalyst precursors.^[^ ^10^ ^]^ However, until now, time‐resolved distinction of phases occurring throughout the suspension aging system remains challenging.

Our hypothesis is that a quantitative understanding of the temporal sequence of the phase transformation processes during aging offers a central tool for optimized process control in the production of a catalyst precursor. The aim of this work is to gain quantitative time‐resolved data of the most relevant aging processes in CZZ catalyst precursor suspensions. These findings need to be discussed in context of the well‐documented CZA aging. Comprehensive analysis of samples collected during the aging period with subsequent washing and drying should give rise to the overall morphological and homogeneity changes and will be measured by scanning electron microscopy (SEM)/energy‐dispersive X‐ray spectrometer (EDXS) and transmission electron microscope (TEM)/EDXS. Further measurements (inductive coupled plasma optical emission spectroscopy (ICP‐OES) and elemental analysis) of the precipitates and of representative liquid filtrate samples will be performed to understand the phase changes in the course of CZZ precursor aging.

## 2 Experimental Section

### 2.1 Continuous Coprecipitation

**Figure 1:**
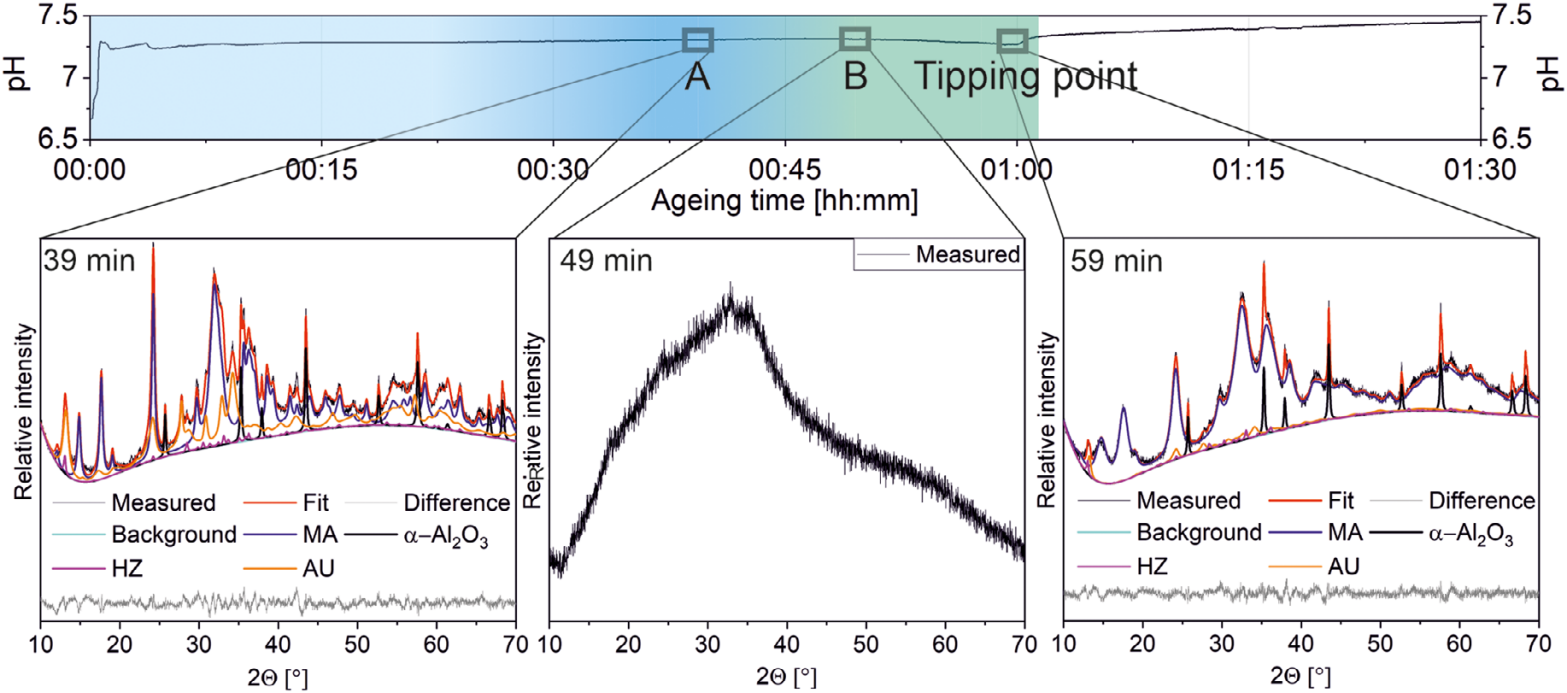
Development of suspension pH and crystalline phase evolution (periods indicated in blue and green) measured by ex situ XRD near the tipping point of a Cu/Zn system with low amounts of Zr (1 mol%) and respective Rietveld refinements. *α*–Al_2_O_3_ is used as internal standard.

The CZZ precursor material was prepared by continuous coprecipitation with subsequent batch aging. The details of the procedure are described elsewhere.^[8c]^ In short, a solution of 127.20 g (0.53 mol) of copper(II)nitrate trihydrate (Cu(NO_3_)_2_·3 H_2_O, Merck, Darmstadt, Germany, 99.5%) 79.52 g (0.27 mol) of zinc nitrate hexahydrate (Zn(NO_3_)_2_·6 H_2_O, Alfa Assar, Thermo Fischer, Kandel, Germany, 99%) and 5.50 g (0.016 mol) of zirconium(IV) oxynitrate hexahydrate (ZrO(NO_3_)_2_·6 H_2_O, Sigma‐Aldrich/Merck, Darmstadt, Germany, 99%) in 3 L of distilled water is mixed under high volume flow (300 mL min^−1^ each metal salts and precipitating agent) with a solution of 1.01 mol L^−1^ NaHCO_3_ at 55 °C using a specific Y‐shaped mixing nozzle. The chosen metal salt composition is in accordance to yield industrially relevant methanol catalyst configurations.^[1a,f]^ The thus created suspension was transferred directly to a double‐jacketed 5000 mL glass vessel and aged at 55 °C under continuous stirring (1000 rpm) until reaching the characteristic pH‐tipping point and 30 min beyond that point. The pH was permanently monitored and its evolution is shown exemplarily in **Figure** **1**. Afterward the aged suspension was filtered and the filter cake was washed with deionized water (≤5 μS cm^−1^) until the filtrate was free of nitrate (< 10 mg L^−1^, *Dosatest Nitrate Nitrite test strips*, *VWR Chemicals*) and its electrical conductivity was below 50 μS cm^−1^. The obtained greenish to turquoise solid was then dried. To follow the course of aging, samples of 25 mL were withdrawn from the aging suspension and immediately filtered to stop further aging of the sample. Subsequently, washing and drying as mentioned above were conducted before analytical investigations.

### 2.2 SEM/EDXS

Field emission scanning electron microscopy (FESEM, Zeiss GeminiSEM 500 equipped with an InLens secondary electron detector and an EDXS, Oxford X‐Max^N^) was used to determine the morphology of the as‐prepared CZZ materials. For each measurement 2 mg of the powdered material was deposited onto a sample holder by a gluey carbon film.

### 2.3 Combustion Analysis for Determination of Light Elements (CHNS)

CHNS analysis was used to investigate the composition of the catalyst, especially detecting the amorphous parts and determining the extent of carbonates or hydroxides as well as hydrates. It was carried out by weighing the sample in tin boats and heating them (1100 °C) in the presence of O_2_ producing the product gases CO_2_, H_2_O, NO_2_, and SO_2_.^[^ ^11^ ^]^ This was followed by controlled reduction of NO_2_ to N_2_ by passing the gas over copper at 850 °C. Finally, the product gas was captured by a column and identified using a thermal conductivity detector. Here, a complete setup called vario EL cube from Elementar was used.

### 2.4 ICP‐OES

For determining the atomic composition (Cu, Zn, Zr, and potential Na), ICP‐OES was applied. Sample preparation takes place by a hydrofluoric acid digestion of 20 mg sample. Digestion was performed with an *Anton Paar Multiwave 3000* microwave oven using HF (40%) in Teflon vessels at 240 °C for 2 h. Subsequent dilution was performed with 0.2 M HNO_3_ Suprapur. For analysis, an Agilent 725 ICP‐OES spectrometer with argon as plasma gas at 15 L min^−1^ and plasma stimulation at 40 MHz, 2 kW.

### 2.5 XRD

X‐ray diffractograms were measured using a Panalytical X’Pert Pro X‐ray diffractometer (Malvern Panalytical GmbH, Kassel, Germany) with Bragg–Brentano geometry and Cu K_*α*_ radiation with a Ni filter. The diffractograms were recorded in the range 5°–80° over a period of 120 min. For quantification of the amorphous amount, 10 wt% *α*–Al_2_O_3_ was mixed with the samples before measurements. The reflections were evaluated using the HighScore Plus software (version 2.2.5) and compared to references from the Joint Committee of Powder Diffraction Standards (JCPDS) database.

### 2.6 Rietveld Refinement

To obtain the composition, particle size and zinc introduction of the samples analyzed by XRD, Rietveld refinement was performed using the open‐source program *Profex* 5.0.2 from Döbelin and Kleeberg.^[^ ^12^ ^]^ The amount of Zn^2+^ in the (zincian) MA phase is determined by a linear fit according to the work of Behrens et al.^[^ ^13^ ^]^ Here, a Vegard‐type behavior was observed until a zinc content of 31 mol%.^[^ ^14^ ^]^

## 3 Results and Discussion

### 3.1 Typical Suspension Aging and Characterization Thereof

**Figure 2:**
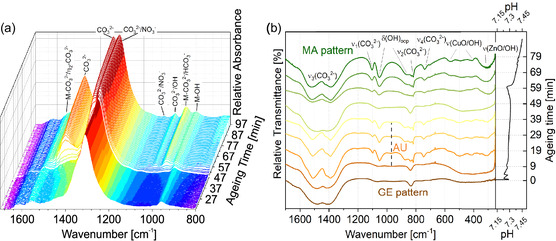
a) In situ and ex situ b) infrared spectra during suspension aging showing typical vibrations for GE, AU, and MA. Copyright CC‐BY 4.0.^[^ ^9^ ^]^

Powder XRD together with Rietveld refinement contributes to a better understanding which crystalline phases are involved in the different aging stages. It has already been reported that the combination of both methods is able to identify the (crystalline) phase composition and also the zinc content in the MA phase.[^8^, ^14^] However, a full distinction between the crystallographically related MA‐ and rosasite (RO) phases is not possible due to broad and overlapping diffraction reflexes (space group for both is P2_1_/a). For sake of simplicity, we consider MA as the target compound in this work, whereas small amounts of RO assumed to be also present are neglected. Therefore, within a certain error margin (see also Figure S1, Supporting Information), the overall crystalline composition can be determined. X‐ray diffraction data exemplarily shown from samples taken during aging demonstrate two main crystallization steps around the pH‐tipping point (Figure 1, see diffractograms; for description of tipping point, see page 4 and following), evidencing that crystalline phase changes are observable and quantifiable. On the other hand, quantification of amorphous phases or of materials with very small particles (≈<10 nm, depending on the material) by XRD is hardly possible. FTIR (**Figure** **2**),^[^ ^9^ ^]^ ICP‐OES, and CHNS analyses are providing additional information on the overall phase composition.

In addition to either copper‐ or zinc‐based single‐metal phases, amorphous sodium‐containing phases occur during the aging period as well as X‐ray amorphous Zr‐based phases (e.g., Zr(OH)_4_, ZrO(OH)_2_, ZrO_2_ × H_2_O; Figure 4).^[^ ^15^ ^]^ As ICP‐OES accurately provides the amount of metals present in the washed precipitate, the formation of such Zr‐based phases alongside Na_2_Zn_3_(CO_3_)_4_ or Na_2_Zn_3_(CO_3_)_4_ × 3 H_2_O (NaZC/NaZCH) is most probable, considering that other Na‐containing species are typically separated (e.g., NaNO_3_) during the filtration/rinsing procedure due to their solubility, which is several orders of magnitude higher (for solubility calculations refer to Supporting Information Chapter 3) than that of the zinc/sodium salts. However, our findings suggest a remainder of additional soluble Na^+^ species probably trapped in the material's pores (see Chapter on page 15). Similar conclusions were made by Zander and co‐workers, that transiently NaZCH is formed in the early stage of suspension aging for CZA materials.^[3h]^ On the other hand, the diffractograms of NaZC and NaZCH^[^ ^16^ ^]^ are very similar, in particular when comparing reflexes in the observable sensitivity range of the authors’ in situ measurements in the mentioned work of Zander et al. This indicates that either NaZC or NaZCH can be present in the early period of suspension aging, even though presence of the hydrate is more likely.^[^ ^16^ ^]^ As these phases are obviously occurring in small amounts or are X‐ray amorphous during CZZ precursor aging (see Figure 1), there is to our knowledge no suitable detection method available to sufficiently distinguish between NaZC and NaZCH during the aging step within this complex mixture. The detection of precipitates of ZrO^2+^ is challenging, too, due to the similar reasons. Specifically, ZrO_2_ is not readily crystallizing in suspension, but forming ZrO(OH)_2_ or similar hydrate species at first, of which a definite speciation is not likely possible without drying and thus changing its hydrated character.^[^ ^17^ ^]^

Overall, quantifying crystalline as well as amorphous substances emerging during aging is achievable by combining ICP‐OES, CHNS analysis with XRD plus Rietveld refinement as well as considering the recent work for FTIR tracking (Figure 2).^[^ ^9^ ^]^

### 3.2 Morphological Changes Throughout Aging of a CZZ Catalyst Suspension

**Figure 3:**
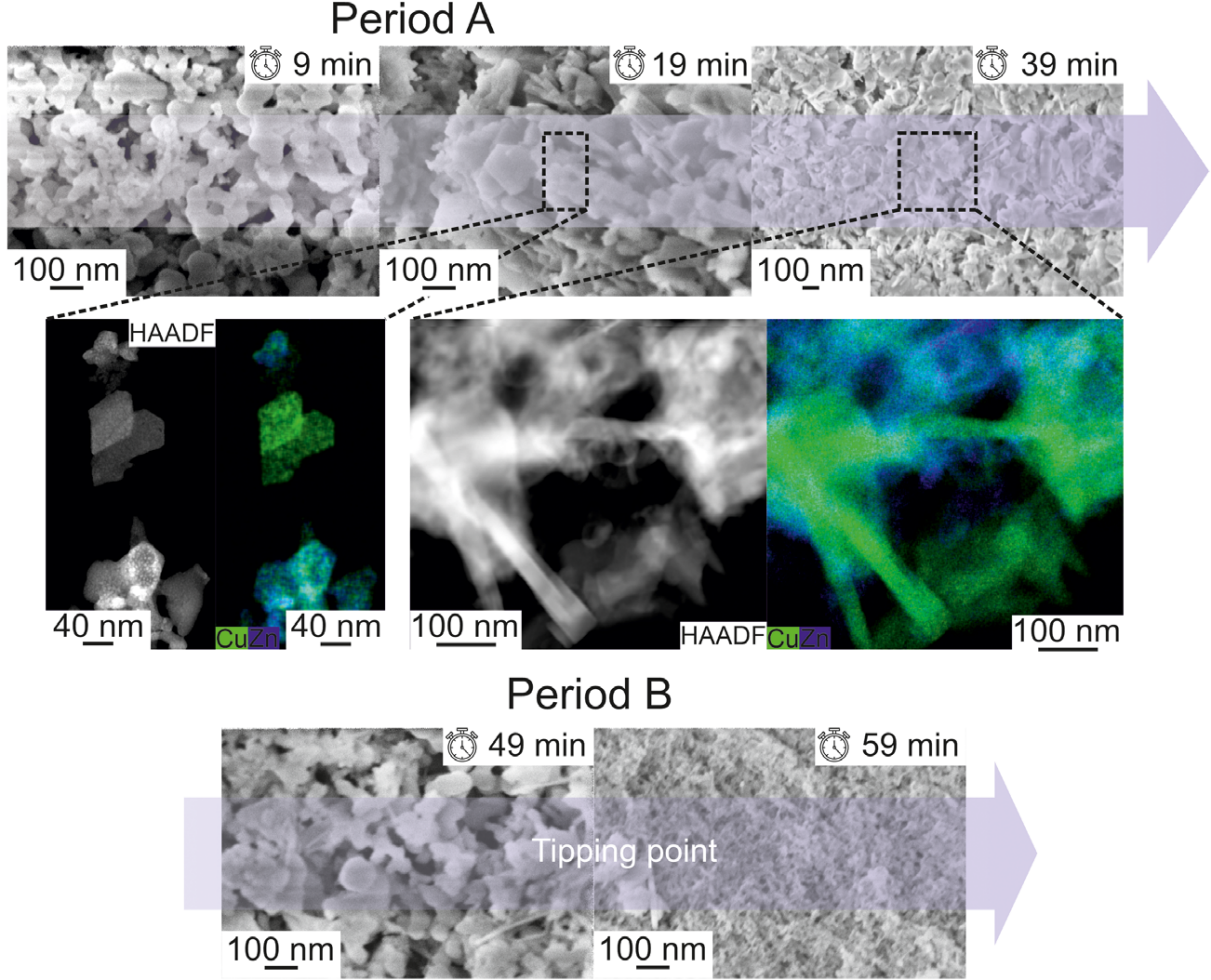
SEM/TEM images as well as EDXS maps with elemental distribution of Cu and Zn. Sampling is indicated corresponding to the aging time. Two crystallization periods (A/B) are observable. After the first one (<39 min), dissolution (49 min), and reprecipitation (59 min) occurs, assumedly.

SEM measurements of samples isolated at defined aging times (samples carefully withdrawn, thoroughly washed and dried), indicate significant changes in morphology (**Figure** **3**), probably due to transient recrystallization (refer to results from XRD measurements, Figure 1).

In detail, after initial nucleation of spherical, X‐ray amorphous particles (<100 nm; at 9 min aging), initial particle growth takes place, resulting in a clearly shaped material with a clear texture and shape (e.g., crystalline, also proven by XRD; Figure S2, Supporting Information) with large crystal‐type platelets (≈100–200 nm; between 19 and 29 min aging, compare: Figure S3–S6, Supporting Information). Afterward, dissolution and partial reprecipitation occur, visible by the formation of a mixed rod‐ and sphere‐shaped, polydisperse looking material (49 min aging). Finally, this material transforms into a fine, anisotropic, nanosized solid (59 min; ≈20 nm). These characteristics differ from the aging in CZA precursors, in which the material is composed of highly shaped rods after aging beyond the pH‐tipping point and where no intermediate recrystallization step was observed during aging. In summary, phases generated in the CZZ system at both, the initial and the final period of aging are similar to those of CZA, however evolving two subsequent crystallization steps.^[3b]^

### 3.3 Changes in Overall Composition: Proposing a Mechanism for the Recrystallization Step

**Figure 4:**
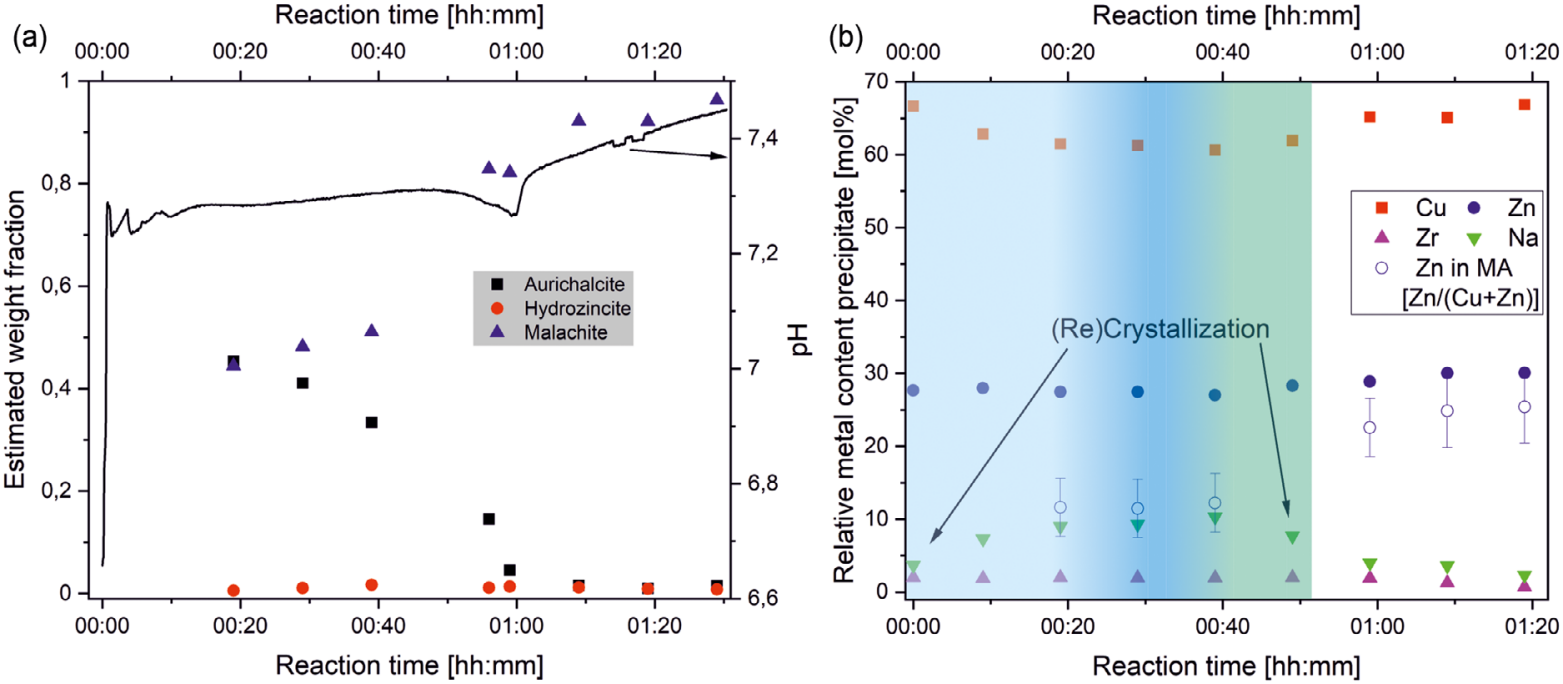
a) Crystalline phase composition obtained by XRD and subsequent Rietveld refinement and suspension pH as function of aging time, with the internal standard *α*–Al_2_O_3_ (subtracted). b) ICP‐OES analysis of precipitates withdrawn during aging.

By investigating the respective samples’ XRD patterns in their chronological order, the overall composition as well as the evolution of the emerging and disappearing solid phases can be determined. In addition, recent work on this system regarding in situ FTIR is considered as well, as this can help understand the formation of poorly crystalline phases (Figure 2).^[^ ^9^ ^]^ Besides changes in XRD analysis, over the course of aging, no significant relative changes in overall metal composition of Cu, Zn, and Zr are observed. On the other hand, the Na content in the filtered solids is varying between 2 and 10 mol% (**Figure** **4**b), most probably due to the formation of NaZC/NaZCH. The occurrence of the latter phases is of importance as they might serve as transitory zinc depot in the solution, supporting the slow (thermodynamic controlled) formation of the targeted Zn–MA phase. However, high amounts of Na‐containing species in the final product would have a negative influence on the quality of the catalyst.^[^ ^18^ ^]^

**Figure 5:**
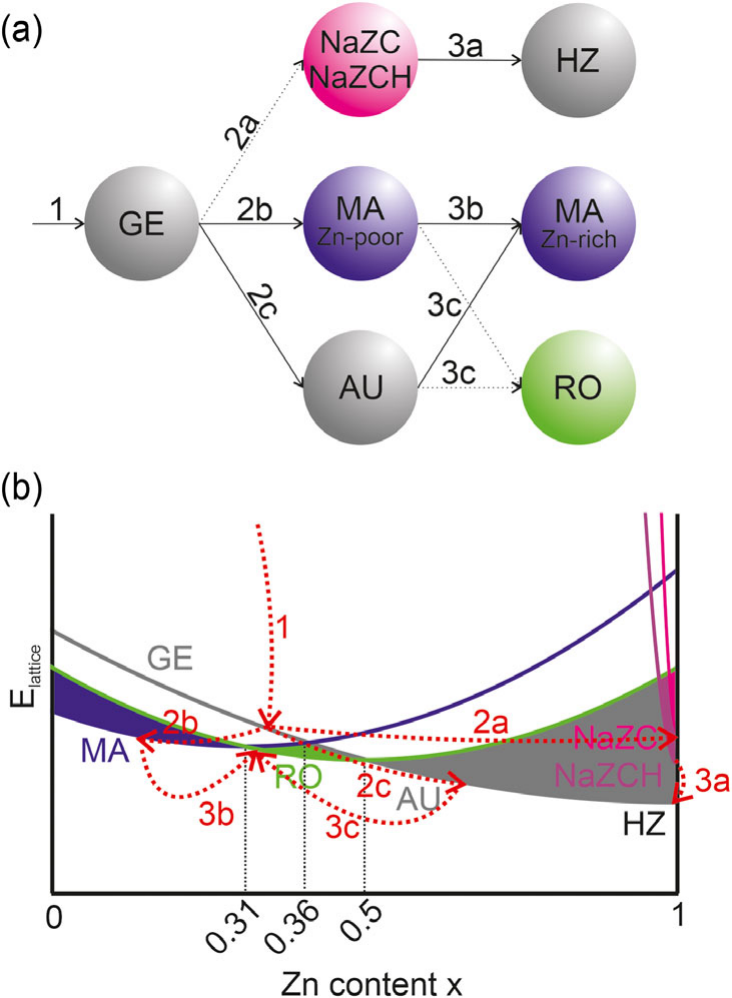
a) Proposed transformation scheme and b) lattice energy of the different occurring phases in the Cu/Zn‐containing bicarbonate system, depending on their zinc content (mol% Zn/[Cu+Zn]). Modified version from Behrens et al. at homogenous defined thermodynamic parameters (temperature, pressure, concentration) applicable to the mechanism described in the text above.

With our experimental approach, kinetically dominated nucleation delivers monodisperse particle seeds as the LaMer–Dinegar model suggests,^[^ ^19^ ^]^ whereas the thermodynamically controlled suspension aging step is separated. Consequently, thermodynamics of aging‐dependent phase transformations have to be considered in detail in order to understand the transient species forming (a modified version of an energetic overview^[^ ^13^ ^]^ can be found in **Figure** **5b** or Figure S8, Supporting Information).

According to Figure 5b and the results discussed above, we describe the general precipitation mechanism for the formation of a CZZ catalyst precursor as follows (overview in Figure 5a): Initially, an amorphous Cu/Zn hydroxycarbonate precipitates (most likely amorphous Zn–georgeite [GE]) together with ZrO(OH)_2_, leaving smaller amounts of Cu^2+^, Zn^2+^, ZrO^2+^, and Na^+^ ions in the solution (Figure 5b, transformation 1). In contrast to Al in CZA materials, ZrO_2_ does not form common phases with Cu^2+^ or Zn^2+^, such as Cu/Zn–Hydrotalcites^[3i]^ or Cu/Zn/Al spinels^[^ ^20^ ^]^ at precipitation conditions. This leads to separate precipitation of ZrO(OH)_2_ that could further influence subsequent phase transformation, which may explain the amorphous nature of the NaZC/NaZCH species, in agreement with the phenomenon of crystallization inhibition caused by impurities.^[^ ^21^ ^]^ As aging progresses, NaZC/NaZCH (according to Figure 4a,b) precipitates together with some aurichalcite (AU; 9–19 min; Figure 5b, transformation 2a and 2b; see also Table S1, Supporting Information). This assumed transformation is supported by time‐resolved ex situ FTIR measurements (e.g., vibration band at 965 cm^−1^, Figure 2) for the aging of CZZ, which were carried out under almost identical process conditions in recent work.^[^ ^9^ ^]^

**Figure 6:**
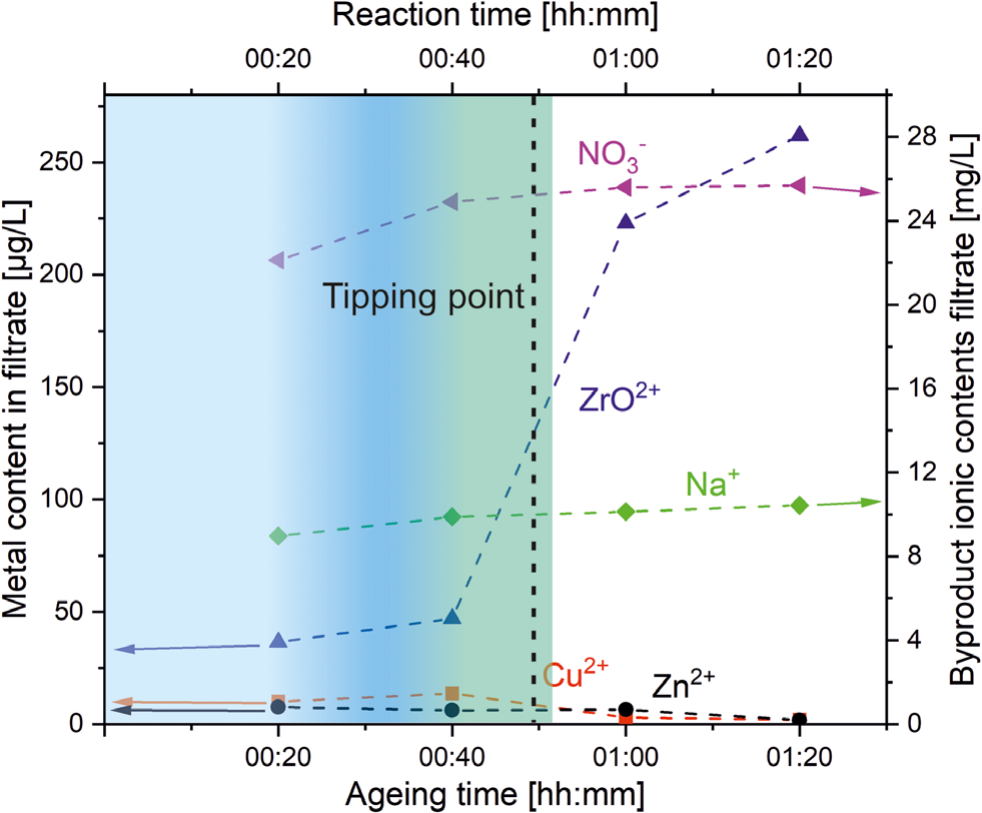
Cu^2+^, Zn^2+^ and ZrO^2+^ concentrations (left *y* axis) as well as Na^+^ and NO_3_^−^ concentrations (right *y* axis) as functions of aging period (see experimental section for details). The tipping point is reached at 49 min. Lines are a guide to the eye only.

In transformation 2b, an increased concentration of dissolved Cu^2+^ (**Figure** **6**) causes a zinc‐poor MA phase to crystallize while concurrently AU dissolves. The transformations occurring during exactly this period are crucial for the subsequent composition of the aged precipitation and allows the introduction of Zn^2+^ into the MA lattice and the emergence of the desirable crystalline Zn–MA phase (19–39 min, Figure 4; Figure 5b, transformation 3b and 3c is beginning). Probably, NaZCH is not stable at these conditions,^[^ ^22^ ^]^ undergoes dissolution and is partly converted to hydrozincite (HZ; Figure 5b, transformation 3a). This increases the concentration of dissolved Zn^2+^, which thus becomes available for incorporation into the MA lattice. At this stage (49 min), a morphologically observed (compare Figure 3) recrystallization occurs during which AU and NaZC/NaZCH gradually dissolve further, while conversely the zinc‐rich MA phase recrystallizes (end of transformation three). At 59 min, ZrO^2+^ concentration rises sharply, underlining the pH‐dependency of its solubility (pH drops rapidly and rises again).

As the Zn–MA recrystallization cannot account for the complete amount of zinc introduced initially, we assume that, at this point, a minor amount of hydrozincite is not transformed.^[^ ^9^ ^]^ Herein, the complex network of phase transitions is described, as shown in Figure 5a. This network is necessary to finally lead to the catalytically most relevant, Zn–MA precursor (with traces of amorphous RO, assumedly), especially including the transient zinc depot Na_2_Zn_3_(CO_3_)_4_ × 3 H_2_O and two different crystallization steps .

## 4 Conclusion

Moving from structural changes, observable with simple and readily available methods, towards a more in–detail analysis, this work contributes to better understanding of the mechanism of the suspension aging, a paramount step for the formation of efficient methanol catalysts.

Considering the data gathered within this study, the generation of CZZ materials using a continuous coprecipitation set‐up shows a marked similarity to the results reported for the well‐documented CZA systems. Still, despite the low amounts of ZrO_2_ incorporated in the desired materials (1 mol%) and the resulting relative similarity to CZA systems, significant differences are noteworthy.

In general, different pH evolution and crystallization tendencies are observed, consequently leading to changes in the overall aging process.

For instance, transient, metastable phases form within the aging step, such as AU and Na_2_Zn_3_(CO_3_)_4_ (x 3 H_2_O), the latter occurring in amorphous form, in contrast to the reports in the aging of CZA catalyst precursors. It is not clear if this Na‐containing phase is completely dissolved until the end of aging, which could imply negative effects on later catalyst performance.

In contrast to CZA, two distinct crystallization steps could be clearly noticed according to SEM and XRD data. These seem to be relevant for the later formation of the target compound zincian MA.

The initially forming zirconium precursor seems to weaken lattice energies in the other phases, as these show altered crystallization tendencies compared to CZA systems. This is tentatively attributed to the absence of any interfering Cu/Al–hydrotalcites and a clear separation of the basic Cu/Zn carbonate phases from the remaining ZrO_2_ × H_2_O/ZrO(OH)_2_, which is clearly not incorporated into Cu/Zn‐containing lattices.

Taken together, the results presented here exemplarily show that considering the aging process as a whole is important for the synthesis CZZ materials. In this regard, synthesis parameters can be studied to avoid Na‐containing species (e.g., low Na^+^ concentration, Zn max. 31 mol% [metals base]). This will support the future scale‐up, optimization of production and let envisage, via the monitoring of specific markers, a growing share of automation in the future synthetic procedure.

## Conflict of Interest

The authors declare no conflicts of interest.

## Supporting information

Supplementary Material

## Data Availability

The data that support the findings of this study are available from the corresponding author upon reasonable request.
