# Supplementary material for: Phase Transformation Processes in Coprecipitated Cu/Zn/Zr Methanol Catalyst Precursors—Insights into Suspension Aging Form Ultrafast Nucleation
Source: Chempluschem. 2025 Jul 9;90(9):e202500284. doi: 10.1002/cplu.202500284 (PMC12435107; doi:10.1002/cplu.202500284)
Supplement: Supplementary file 1 — Supplementary Material [file CPLU-90-e202500284-s001.pdf]

– SUPPORTING INFORMATION –

Content:

1. **XRD of timed samples and error margin of XRD & *rietveld***
2. **TEM investigations during ageing time (HAADF-STEM, STEM/EDX)**
3. **Calculation of maximum $NaNO_3$ content in timed samples**
4. **Changing amount of sodium throughout ageing**
5. **Thermodynamics in the Cu/Zn-bicarbonate system**

XRD of timed samples and error margin of XRD & *rietveld*

Refined Parameters		
Parameter	Value	ESD
Global GOALs		
QabsMalachite	0.7440	0.0570
Qabsaurichalcite	0.1310	0.0110
QabsHydrozincite	0.0100	0.0006
QabsAmorph	0.1140	0.0590

Figure S1. Error margin of the weight fractions of a sample fitted with rietveld refinement. ESD: Estimated standard deviation.

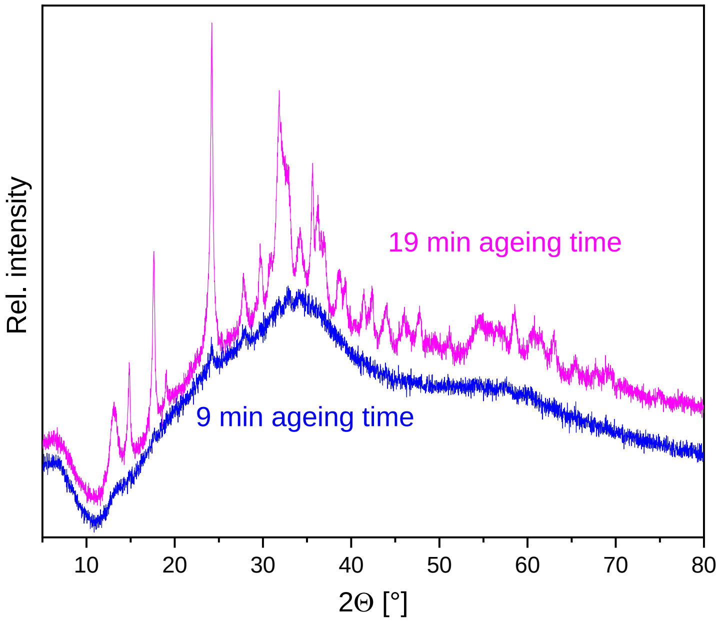

Figure S2. Initial crystallization described in the main text, observable by changing reflex intensity in XRD.

TEM investigations during ageing time (HAADF-STEM, STEM/EDX)

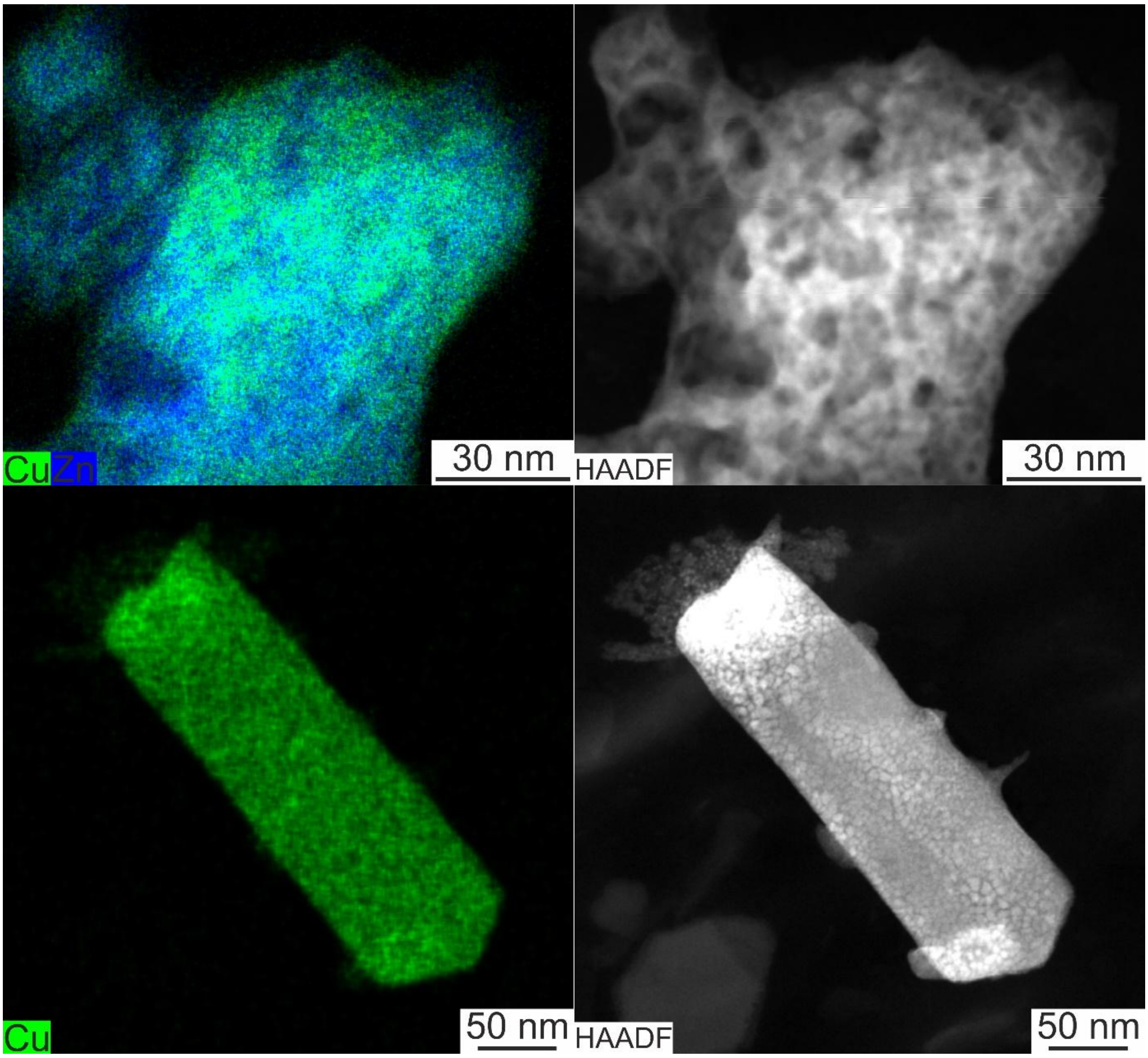

Figure S3. TEM investigation after 19 min ageing time. Elemental distribution determined by EDXS (left) and HAADF-STEM (right).

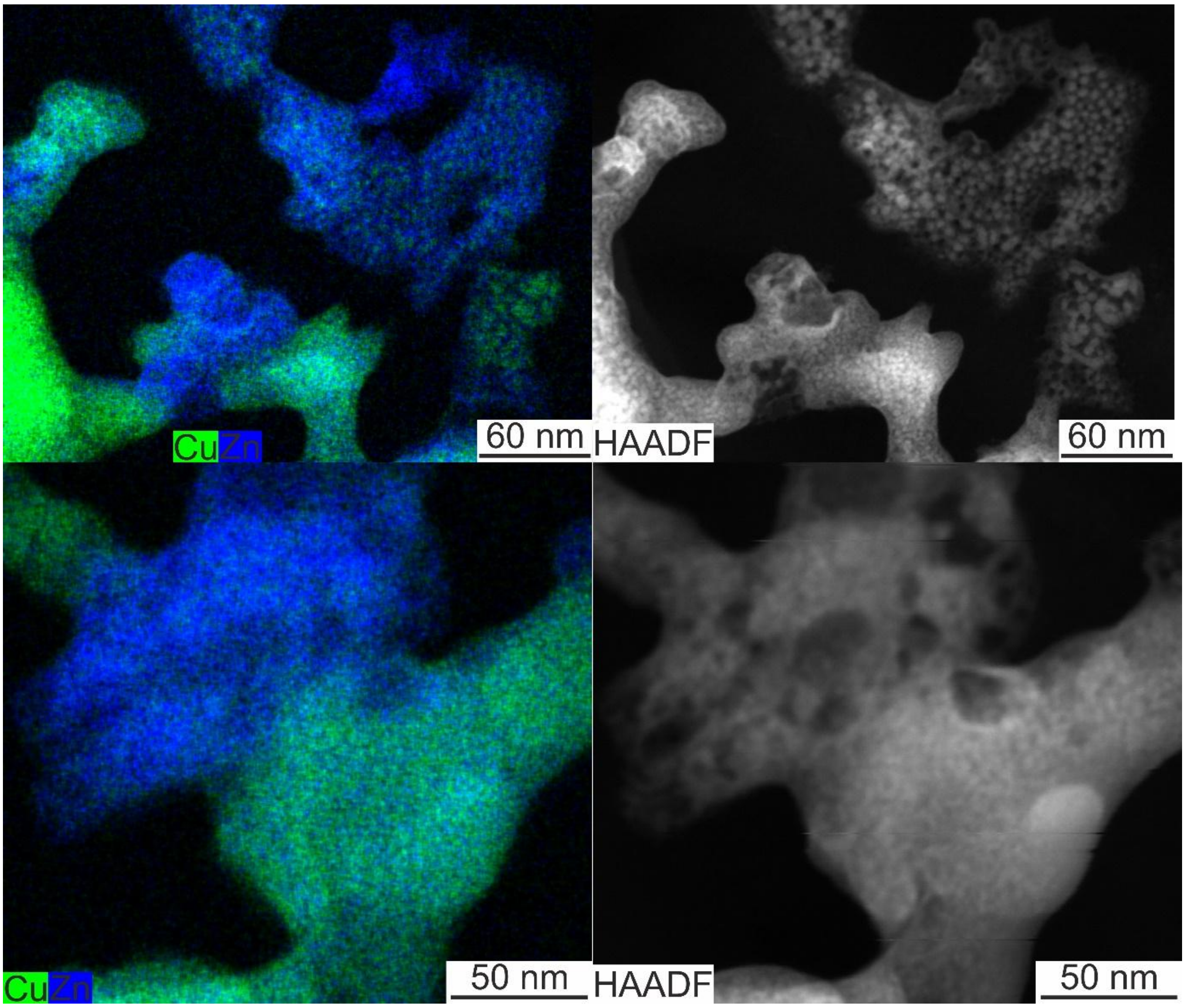

Figure S4. TEM investigation after 39 min ageing time. Elemental distribution determined by EDXS (left) and HAADF-STEM (right).

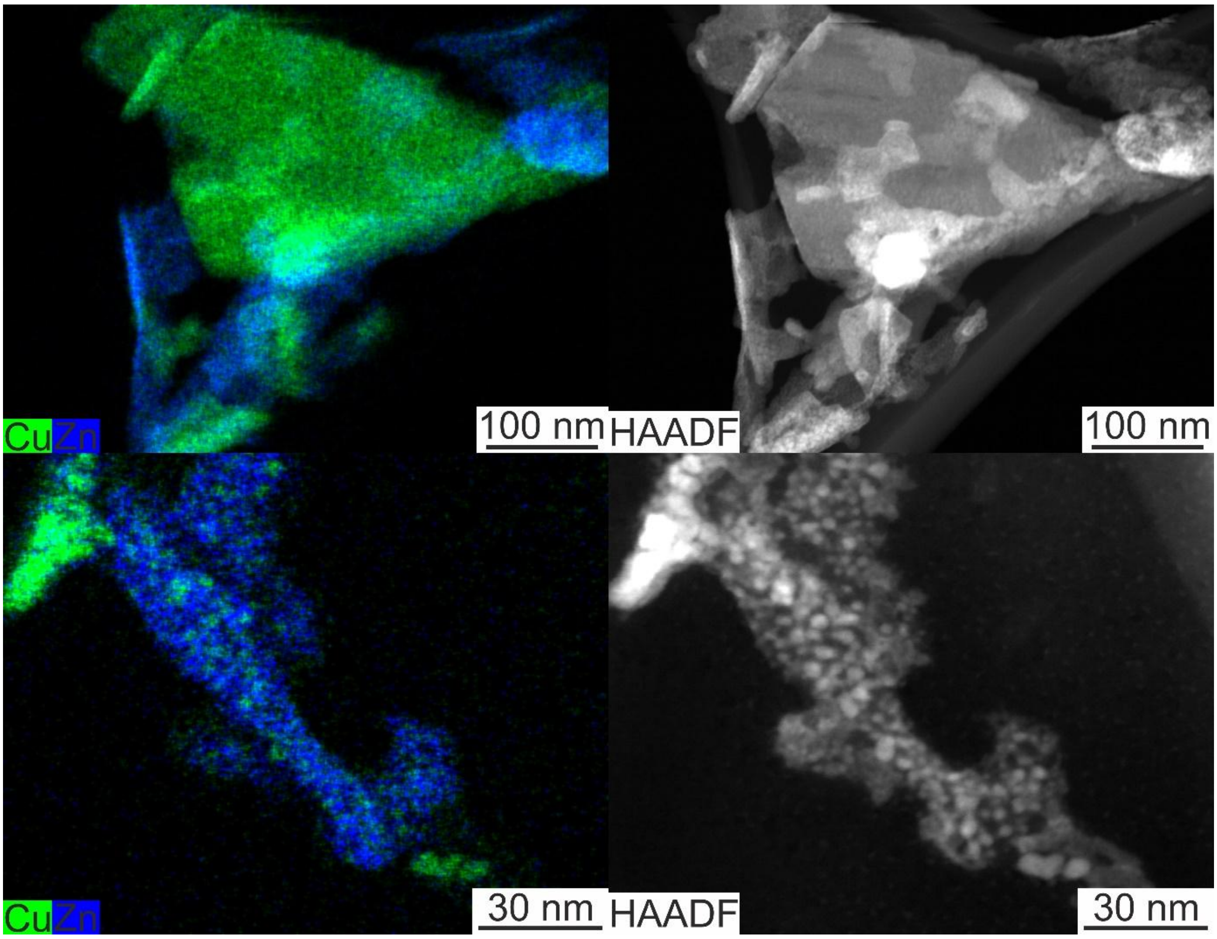

Figure S5. TEM investigation after 49 min ageing time. Elemental distribution determined by EDXS (left) and HAADF-STEM (right).

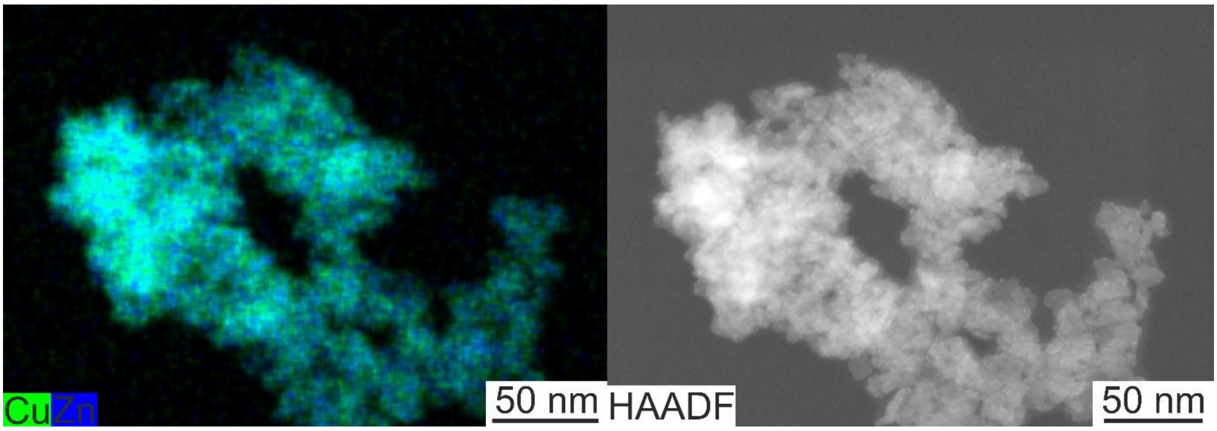

Figure S6. TEM investigation at 99 min ageing time. Elemental distribution determined by EDXS (left) and HAADF-STEM (right).

Calculation of maximum $NaNO_3$ content in timed samples

To calculate the maximum amount of $NaNO_3$ and Na respectively, the *Kohlrausch*'s squareroot law[1] together with conductivity measurements from the literature[2] have been employed. From the *Kohlrausch* fit of molar conductivity vs. squareroot of $NaNO_3$ concentration (Figure S7), conductivities can be attributed to specific concentrations under the assumption, that dissociated $NaNO_3$ makes up for the total conductivity at the end of washing (see also Figure 9). As all samples of precipitates were thoroughly washed with deionized water until the conductivity of the filtrate was below 50 µS/cm, the following calculation leads to the maximum amount of Na being possibly present:

$$\omega_{Na} = n_{m,Na} * 100$$

$$with \qquad n_{m,Na} = \frac{c_{Na}}{\beta_{Solids}} \qquad and \qquad c_{Na} = c_{NaNO3} = \frac{\kappa}{a} - b$$

ω_{Na}: Mass content of Na [wt% Na]

$n_{m,Na}$: Mass-based molar concentration of Na [mol_{Na}/g_{solid}]

c_x: Concentration of x [mol/L]

β_{Solids}: Mass concentration of solids in suspension [g/L]; in this case 11 g/L

κ: Maximum conductivity of filtrate [µS/cm]; in this case 50 µS/cm

a: Slope of the fit of *Kohlrausch*'s law

b: Y-axis intersection of the fit of *Kohlrausch*'s law

From the mentioned fit and the described calculation, the maximum Na mass content in the precipitate is 0.1 wt%.

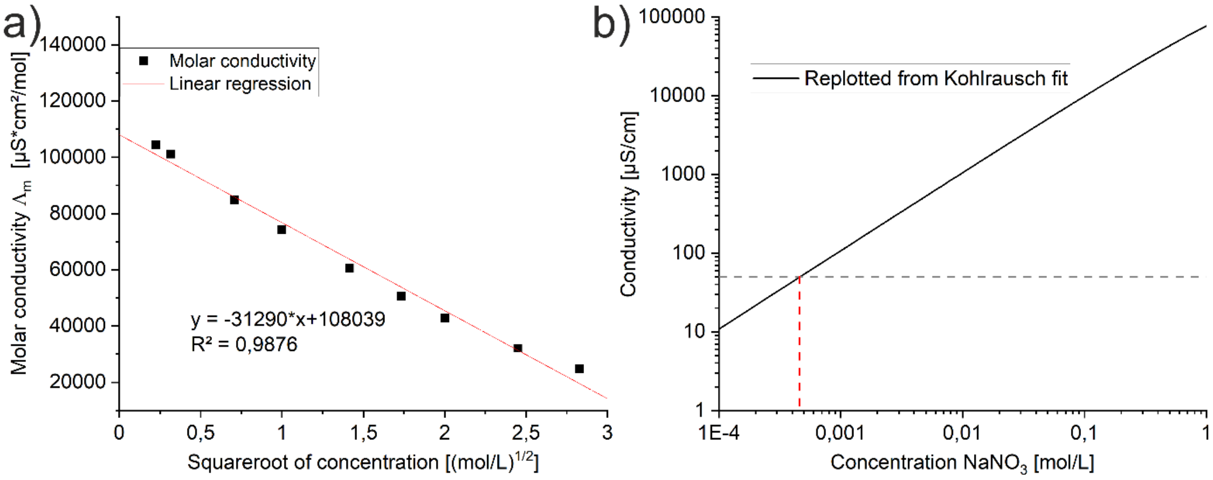

Figure S7. a) *Kohlrausch* fit of the data obtained from literature. b) Replot of this fit after conversion of molar conductivity to conductivity. The horizontal line marks the conductivity below which precipitates have been washed whereas the vertical line in red marks the respective $NaNO_3$ concentration.

Changing amount of sodium throughout ageing

As the Na^+ found in the precipitate of timed samples is ascribed to NaZCH minus the threshold of residual Na^+ (see below and chapter 2), the amount of free Zn [1-(Zn_{NaZCH}/Zn_{Total})] in the solid can be calculated. However, as mentioned in the main text, NaZCH is assumed to dissolve completely till the end of the suspension ageing. The remaining Na content may be attributed to other Na-species in the solid’s pores even after washing.

Table S1. Content of different compound in the first aging period, depending on ageing time. Sodium, NaZCH, the respective Zn content in NaZC(H) in comparison to the total Zn amount (rounded).

Ageing time	Na (wt%)	Na without residual (wt%)	NaZCH (wt%)	Zn_{NaZCH} (wt%)	Zn_{NaZCH}/Zn_{Total} (%)
**0 min**	0.7	0.2	2.3	0.9	5.7
**9 min**	1.5	1.0	11.7	4.3	18.3
**19 min**	1.9	1.4	16.3	6.0	28.6
**29 min**	1.9	1.4	16.3	6.0	31.2
**39 min**	2.2	1.7	19.8	7.3	35.3
**49 min**	1.6	1.1	12.8	4.7	22.1
**59 min**	0.8	0.3	3.5	1.3	11.1
**69 min**	0.7	0.2	2.3	0.9	4.1
**79 min**	0.5	0	0	0.0	0.0

Thermodynamics in the Cu/Zn-bicarbonate system

Shown are potential steady-state relations suggested by Behrens *et al.*[3]

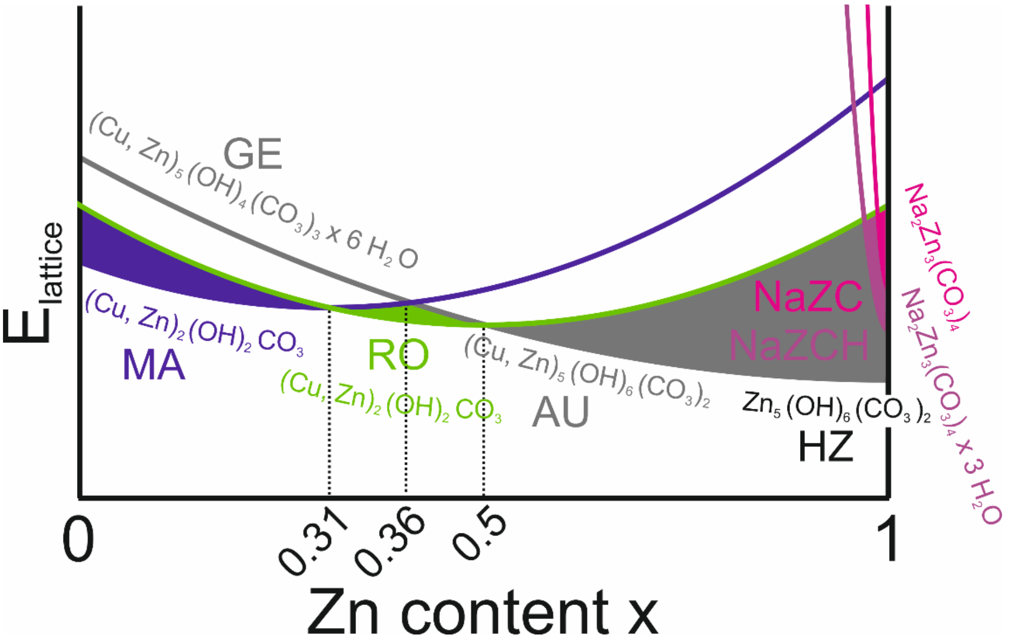

Figure S8. Potential steady state relations of the different occuring phases in the Cu/Zn-containing bicarbonate system, depending on their Zn content (mol% Zn/[Cu+Zn]). Modifed version from Behrens *et al.*[3]

References

[1] E. Katz, *Electrochemical Science Advances* **2022**, *2*. 10.1002/elsa.202160008

[2] T. Isono, *Journal of Chemical & Engineering Data* **1984**, *29*, 45–52. 10.1021/je00035a016

[3] M. Behrens, F. Girgsdies, *Zeitschrift für anorganische und allgemeine Chemie* **2010**, *636*, 919–927. 10.1002/zaac.201000028
